# Structure of African swine fever virus p15 reveals its dual role for membrane-association and DNA binding

**DOI:** 10.1007/s13238-020-00731-9

**Published:** 2020-05-25

**Authors:** Dan Fu, Dongming Zhao, Wei Zhang, Guangshun Zhang, Mingyu Li, Zheng Zhang, Yuhui Wang, Dongdong Sun, Peng Jiao, Cheng Chen, Yu Guo, Zihe Rao

**Affiliations:** 1 State Key Laboratory of Medicinal Chemical Biology and College of Pharmacy, Nankai University, Tianjin, 300350 People’s Republic of China; 2 State Key Laboratory of Veterinary Biotechnology, Harbin Veterinary Research Institute, Chinese Academy of Agricultural Sciences, Harbin, 150001 People’s Republic of China; 3 School of Life Sciences, Tianjin University, Tianjin, 300071 People’s Republic of China

**Dear Editor,**

African swine fever (ASF) is a highly contagious disease of domestic pigs caused by African swine fever virus (ASFV), with mortality rates approaching 90%–100%. The first ASF case was described in 1921 in Kenya. Over the past decades, ASF has spread from Sub-Saharan Africa to Europe and Asia, and pose a huge threat to global food safety. Since the first ASF case reported in August 2018, more than 10 million pigs had been culled in China at an enormous economic loss. The risk of ASFV mutating to infect humans is extremely low. However, ASF not only threatens food security but also severely strikes the world economy in many fields, such as the global supply of the blood thinner heparin, most of which is produced from pigs. Hence, ASF is a strictly monitored infectious animal disease by OIE (World Organization for Animal Health), unfortunately, there is no approved drug or vaccine against this lethal viral disease.

ASFV, the pathogen of ASF, is the only member of the family *Asfarviridae*, which belongs to the group of nucleocytoplasmic large DNA viruses (NCLDVs), and is the only known DNA arbovirus to date (Iyer et al., 2001). The approximately 250 nm in diameter of ASFV is a complex multilayer structure: the external envelope of extracellular virus derived from the plasma membrane when the progeny virus buds through the host plasma membrane; the icosahedral protein capsid constituted by major capsid protein p72 and over 50 minor capsid proteins; the inner lipid envelope originated from the endoplasmic reticulum; the 30 nm thick matrix-like core shell and the central DNA-containing nucleoid. Such an elaborate stable architecture of ASFV particles provides a facility in the environment for virus long-standing exist. The icosahedral capsid layer and core shell layer jointly protect the genome from host cell nuclease degradation. It is worth noting that both intracellular and extracellular ASFV particle forms are infectious (Andres et al., 2001), indicating a multifunctional role of the virus particle inner protein. Recent Cryo-EM studies on ASFV particle have greatly improved our understanding of the icosahedral protein capsid layer architecture (Liu et al., 2019; Wang et al., 2019; Andres et al., 2020). The icosahedral capsid of ASFV is composed of 2,760 copies of the double jelly-roll major capsid protein p72 and resembles other NCLDVs. The core shell layer, which accounts for about 30% of the virion protein mass, is formed underneath the inner lipid envelope and surrounds the central electron-dense nucleoid. Early studies have indicated that the mature products p150, p37, p34, p14, and p5 derived from polyprotein pp220 and p35, p15, and p8 derived from polyprotein pp62 are the basic components of the core shell (Simon-Mateo et al., 1997; Andres et al., 2002; Suarez et al., 2010). Interestingly, among all the identified components in ASFV core shell, only p15 contains about 26% β-strands in contrast to other medium-size core shell proteins predicted by Phyre2, while other proteins are predicted to be mainly consisted by helix (Andres et al., 2020). The recent investigation on virus particle unveils that the core shell which is assembled by pseudo-hexameric capsomers, displays a different triangulation number (*T* = 19) in comparison with the outer capsid (*T* = 277). However, the detailed architecture of core shell structural proteins remains elusive due to low resolution (9 Å or lower) (Wang et al., 2019; Andres et al., 2020). The lack of structural and functional information limited our understanding of the assembly mechanism of the virus core shell.

In this study, we present the crystal structure of mature protein p15 (159 amino acid residues) at 2.6 Å and reveal its dual role for membrane-association and DNA binding. We expressed the recombinant p15 with a hexahistidine tag at the C- terminus in the *Drosophila* S2 expression system. The p15 protein purified by gel filtration chromatography exists three states in solution, eluted as the monomer, dimer (as the predominant state), and hexamer according to the elution volume. We further confirmed this result by analytical ultracentrifugation (AUC) method and native PAGE assay (Fig. 1A). Due to the lack of a homologous model, the crystal structure of p15 is finally determined using the single-wavelength anomalous dispersion (SAD) method by the selenium derivative. The p15 model was refined to 2.6 Å with the *R*_factor_ and *R*_free_ of 18.12% and 21.81%, respectively (Table S1).

**Figure 1:**
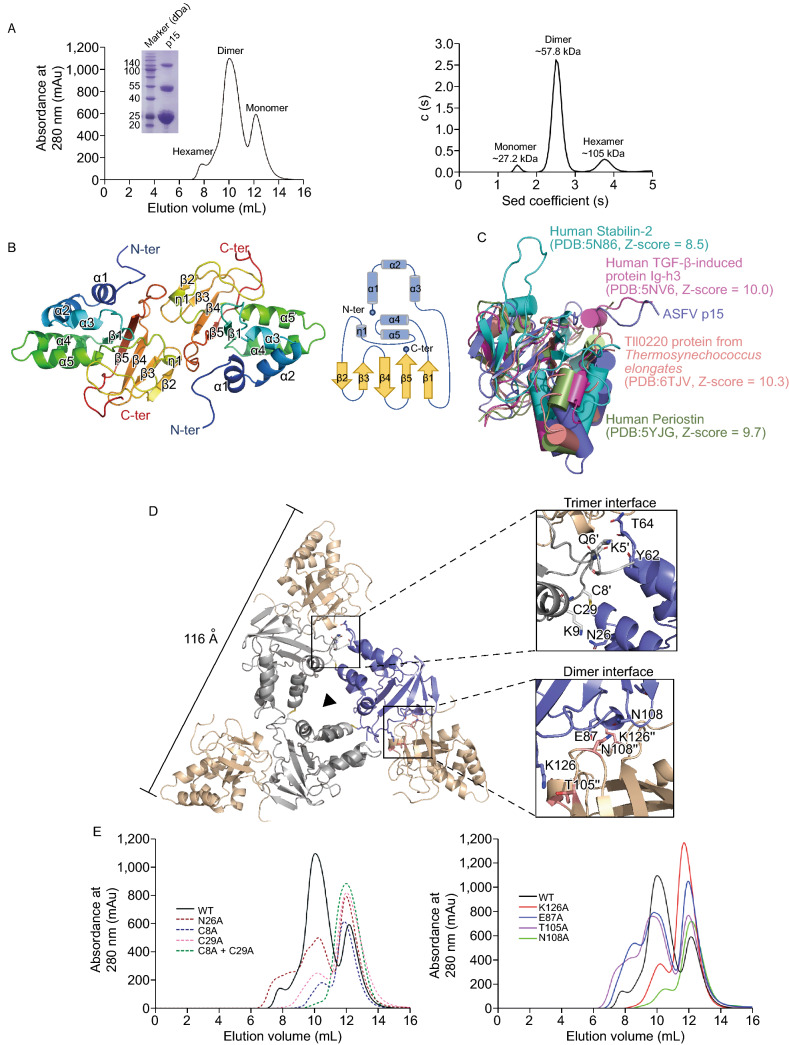
**The overall structure of ASFV p15**. (A) Size-exclusion chromatogram of ASFV p15 protein. Three distinct p15 peaks corresponding to hexamer, dimer and monomer,respectively. The states of p15 in solution are confirmed by Native-PAGE assay and AUC assay. The calculated molecular weights corresponding to each peak in AUC are labelled above the curve. Sed stands for sedimentation. (B) The crystal structure of ASFV p15 dimer is shown in cartoon together with the topology diagram of the monomer. The secondary structure elements are coloured in rainbow and numbered consecutively. In the topology diagram, β-strands are represented as gold arrows, while α-helices are shown as blue cylinders. (C) Superposition of p15 (Slate) with Human stabilin-2 (Cyan), Human TGF-β-induced protein Ig-h3 (Pink), Tll0220 protein from *Thermosynechococcus elongates* (Salmon), and Human Periostin (Palegreen). All the structures are shown as ribbon. (D) Top view of the ribbon diagram of the ASFV p15 hexamer. Crystallographic symmetry generates the hexametric “three-blade propeller”- like structure, comprised of three dimers. The side length of the hexamer is labelled. The detailed interactions within the trimer interface and dimer interface are shown at the right panel, respectively. The residues on the interface are labelled and shown as sticks. (E) Mutational analysis of the interface by SEC. The absorbance at UV280 nm of proteins is coloured in different coloured lines

The space group of p15 crystal is determined to be *P*2_1_3, one asymmetry unit contains two molecules, and each molecule is composed by five α-helices, one 3_10_-helix and five β-strands, adopted as a wedge-like structure, with an α-helices bundle located on one side and a β sheet fold on the other side. The triangle α1-α2-α3 and V-shape α4-α5 are connected by the β1 strand, whereas the β5 strand at the center is in parallel with β1 strand. The 3_10_-helix η1, located at the edge of the compact fold, connects the α5 and β2 strand. The β2/β4 strands are antiparallel with β1/β5/β3 to form the stable β sheet structure (Fig. 1B).

Interestingly, despite the failure of three-dimensional structure prediction based on primary sequence, the PDB DALI research (Holm and Sander, 1995) renders some novel information. According to the matches against PDB25 database, the p15 protein shows the highest degree of structural similarity with certain proteins, including Tll0220 protein from *Thermosynechococcus elongates* (with Z-score of 10.3 and RMSD of 2.7), human TGF-β-induced protein Ig-h3 (with Z-score of 10.2 and RMSD of 3.0), human periostin (with Z-score of 9.7 and RMSD of 3.9), and human stabilin-2 (with Z-score of 9.2 and RMSD of 2.8). Although these proteins varied from their distinct species and function, all of them share the same protein fold, called the Fasciclin 1 (FAS1) domain (Fig. 1C). The FAS1 domain is an ancient structural motif in extracellular proteins present in animals, plants, fungi, eubacteria, and archaea (Seifert, 2018). By the interface constituted by the β sheet fold, FAS1 domain accommodates multiple interactions. To our knowledge, it is the first time that the FAS1 domain is discovered in viral structural protein. This finding not only expands the knowledge of FAS1 domain distribution in different species but also implies the unique function of ASFV p15.

As we described previously, the recombinant p15 protein existed as higher oligomerization state in solution, when more symmetry molecules were generated, a homohexamer composed of three dimers can be clearly identified. The p15 hexamer resembles a three-blade propeller structure, with a side length of 116 Å (Fig. 1D). Three p15 molecules from each dimer assemble along the triple-axis in the center and form the core of the hexametric center, mainly by the disulfide bond connection within adjacent dimers. For the trimer interface, there are mainly two types of interaction force: disulfide bonds as the major force, provided by the residues C29 and C8’; then stabilized by multiple Vander Waals interaction and hydrogen bond, formed by the residues T64, Y62 from one dimer and K5’, Q6’ from the other dimer. It is worth noting that the residues located in N-terminal play the major role for interaction within trimers, subsequent Cys-Ala mutagenesis experiments further support our observations (Fig. 1E, left panel). Besides, we also notice that along with the disruption of the hexamer, the dimer transformed into monomer (Fig. 1E, left panel). Meanwhile, for the interactions in dimer interface, the oligomer is mainly stabilized by electrostatic interactions between two β sheets: the residues E87 with K126’, N108 with N108’, K126 with T105’. The Ala mutagenesis experiments of the residues E87, K126, N108, and T105 further confirmed their respective roles for dimer interaction (Fig. 1E, right panel).

The FAS1 superfamily includes members from all phylae, and FAS1 or FAS1-like structures are present in many secreted or membrane-anchored proteins. For p15 hexamer, three monomers are connected by disulfide bonds to form the hexamer core, and the other three monomers interact with their respective counterpart via the β sheet interface. This unique hexamer structure promotes us to further investigate its role during virus infectious cycle. Note that p15 and other components in the core shell are responsible for the association with virus nucleoid in the early phase of virus packaging, hence mediate the DNA nucleoid layer and inner membrane layer of ASFV particle, we speculate that ASFV p15 protein may possess some properties like the virus matrix protein. By homology searching from the protein data bank and some related literature, we found that this “three-blade propeller” shape of p15 shares structural homology with the matrix protein from *retroviruses*, such as simian immunodeficiency virus (SIV) matrix antigen (Rao et al., 1995) and Human immunodeficiency virus (HIV) matrix (Hill et al., 1996), despite their low primary sequence similarity. By calculating the electrostatic potentials of ASFV p15 hexamer and SIV matrix trimer with APBS tools, we located several interesting regions of these two oligomers. For the convenience for interpretation, we termed two sides of the hexamer as “Side A” and “Side B”, respectively. “Side A”, which is featured by a distinct negatively charged region (encircled by a red circle in Fig. 2A) near the hexamer core as SIV matrix trimer, contains the positively charged residues K52, K49, K126, K104, and K5 in each monomer. For “Side B”, we can also locate positive charged R131, R132, K38, and K9 on each of the monomer. By carefully examining the unique feature of these two proteins, we can visualize some obvious differences (Fig. 2A). Firstly, for the SIV matrix trimer, most of the positively charged surface regions are located at the “Side A”, in contrast to the evenly distributed on two sides for ASFV p15 hexamer; secondly, most of the basic residues are located on the edge of SIV matrix trimer, while basic residues are distributed dispersedly in both sides of ASFV p15 hexamer; thirdly, at the hexametric core along the triple symmetry axis, “Side A” possesses acidic residues D18, D20, and E23, while “Side B” possesses the basic residues K9, R131 and R132 of ASFV p15 hexamer. In contrast, the acidic residues around the triple symmetry axis are mostly located at Side A for the SIV matrix, with no significant positive charge core on the other side.

**Figure 2:**
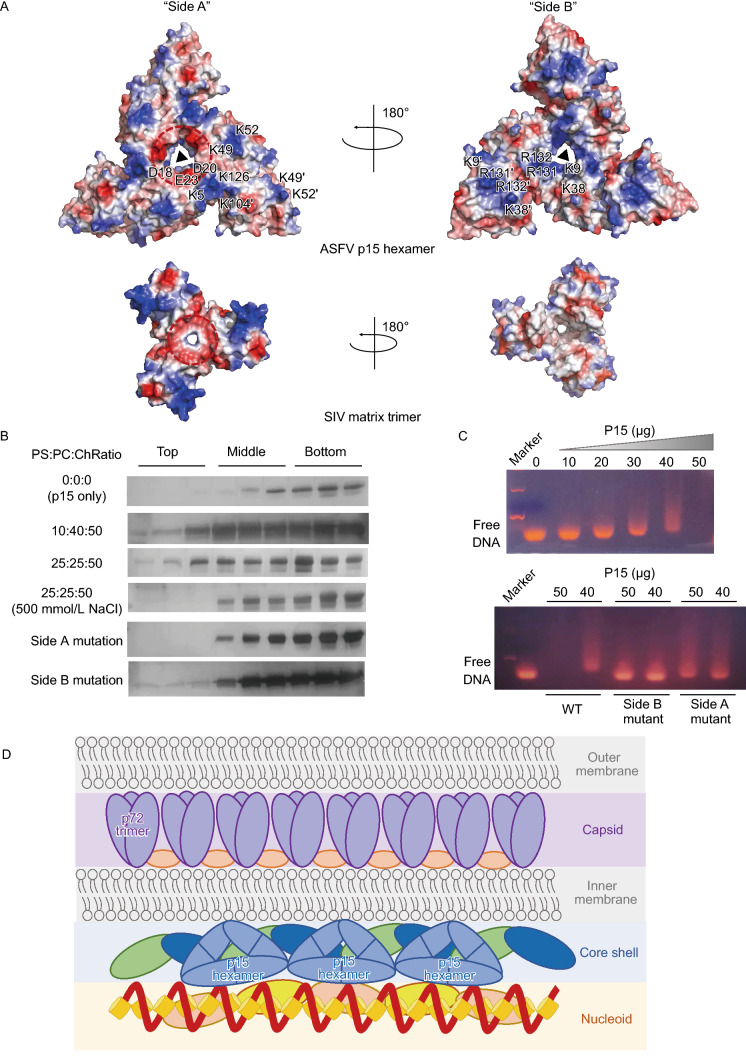
**The dual role for membrane-association and DNA binding of ASFV p15.** (A) The solvent-accessible surface of ASFV p15 hexamer and SIV matrix trimer are coloured according to electrostatic potential ranging (±5 kT/e) from blue (basic or positively charged) to red (acidic or negatively charged). The front view (“Side A”) and back view (“Side B”) of ASFV p15 hexamer and SIV matrix trimer are displayed. The critical residues on ASFV p15 surface are labelled. The negatively charged regions of ASFV p15 hexamer and SIV matrix trimer are highlighted by a red circle. (B) Liposome flotation experiments of ASFV p15 protein. Interaction of wild-type p15 and mutants with liposomes are tested in flotation experiments against different lipid compositions and NaCl concentrations. Top, Middle, and Bottom fractions of the discontinuous gradient were analyzed by SDS-PAGE and stained with rapid silver staining kit. PS:phosphatidylserine, PC: phosphatidylcholine, Ch: cholesterol. (C) EMSA assay of ASFV p15 wild-type protein and mutants binding to dsDNA. The amount of p15 protein is labelled on the top, unbounded free DNA are labelled on the left side. (D) A possible model illustrating the location and function of p15 hexamer in ASFV core shell structure. The five layers of ASFV are labelled on the right side. The p15 hexamer is shown as a blue triangle. The other proteins are shown as different colour and size shapes. The DNA-genome is shown as a dark red spiral

The matrix protein from *retrovirus* and *orthomyxovirus* has been shown to bind negatively charged liposomes and the lipid envelope. Here, we firstly tested the possibility of ASFV p15 hexamer for binding with phospholipids by liposome floatation experiments, following a previously described protocol with some modification (Ruigrok et al., 2000). The ASFV p15 hexamer solely cannot be detected in the top fraction, in comparison, when incubated with liposomes of different compositions, the p15 hexamer appears at the top fraction (Fig. 2B). The result confirmed the ability of ASFV p15 to interact with phospholipids, with the preference for negatively charged phosphatidylserine. Besides, the high ionic strength (500 mmol/L NaCl) significantly prevents the lipid-binding (Fig. 2B), suggesting that ASFV p15 lipid binding is mainly mediated by electrostatic interactions. This result is in consistent with previously reported influenza virus M1 protein (Ruigrok et al., 2000). To further validate which side plays the major role for liposome binding, we design the mutants experiment on each side to explore their individually binding affinity with the lipids. The result clearly shows that the lipid-binding ability is totally abolished when the basic residues in “Side A” are mutated into Ala residues. In contrast, the mutants in “Side B” still retains some binding ability with liposome (Fig. 2B). These results suggest that positively charged residues on “Side A” likely play an important role in facilitating host cell membrane recruitment and association. Similar phenomena are also reported in *retrovirus* and *orthomyxovirus* matrix proteins (Rao et al., 1995; Hill et al., 1996; Sha and Luo, 1997; Ruigrok et al., 2000).

Moreover, it is also worth noting the difference between ASFV core shell and matrix layer from *orthomyxovirus* or *retrovirus*: ASFV core shell is a 30 nm thick layer comprised of multiple structural proteins, compared with the relatively simpler protein interaction by virus matrix protein. Besides, during the assembly and packaging process, the virus genome DNA, or nucleoid will recruit pp220 and pp62 derivatives to form core shell (Simon-Mateo et al., 1993; Simon-Mateo et al., 1997; Andres et al., 2001; Andres et al., 2002), however, there is no evidence directly indicate which component of ASFV core shell interacts with the viral DNA genome. Hence, to test the nucleic acid binding affinity of p15 hexamer, we detected its binding activity with dsDNA by EMSA assay. The result shows that p15 hexamer possesses the binding affinity with the DNA segments derived from the ASFV genome (Fig. 2C, upper panel). Since we have shown that the p15 hexamer binds with the inner lipid membrane via the interface from “Side A”. Therefore, we speculate that the “Side B” may involve in the binding with DNA segments. To further confirm this speculation, we use the same mutants in liposome floatation experiments to perform the EMSA assay. Compared with the control group, the “Side B” mutants completely abolish the ability for the binding with DNA segments, while “Side A” mutant still retains some binding ability with the dsDNA probe (Fig. 2C, lower panel). Therefore, by combining the results from liposome floating and EMSA assay of ASFV recombinant p15 hexamer, we raise the hypothesis that ASFV p15 may play the dual role for membrane association and DNA binding, via the interface from “Side A” and “Side B”, respectively. We speculate that this unique dual-function should play a vital role in ASFV morphogenesis.

ASFV particle is a very complex structure with five layers: the outer membrane, the icosahedral protein capsid, the inner lipid envelop, the core shell, and the genome-containing nucleoid (Simon-Mateo et al., 1997; Liu et al., 2019; Wang et al., 2019; Andres et al., 2020). Based the structural and functional investigation on ASFV p15 protein, we propose a possible model for illustrating the location and function of p15 hexamer in ASFV core shell structure (Fig. 2D): the icosahedral protein capsid containing major capsid component p72 and minor capsid factors locates between the outer membrane and the inner membrane; the thick core shell binds inner membrane and encapsidates the genome-containing nucleoid through interactions with structural proteins derived from pp220 and pp62. Given that core shell is a 30 nm thick layer comprised of multiple structural proteins, it is highly possible that p15 interacts with other components during the virus assembly process and facilitates the stabilization of the mature virus particle. Of course, more evidence from the high-resolution structure and functional assay of core shell proteins are needed to further elucidate this vital question.

In this work, we present the high-resolution crystal structure of ASFV core shell component p15. The p15 monomer resembles the FAS1 domain, an ancient structural motif structure. Moreover, p15 form a higher-organized hexamer structure via disulfide bond and electrostatic force within adjacent monomers in solution. We further map the regions which are responsible for membrane association and DNA binding, respectively, reveal its dual-role in the virus infectious cycle. Generally, our work may be one of the important steps toward the understanding of ASFV core shell assembly.

## Electronic supplementary material

Below is the link to the electronic supplementary material.

Supplementary material 1 (PDF 240 kb)
