# Supplementary material for: Structure of African swine fever virus p15 reveals its dual role for membrane-association and DNA binding
Source: Protein Cell. 2020 May 25;11(8):606–12. doi: 10.1007/s13238-020-00731-9 (PMC7381529; doi:10.1007/s13238-020-00731-9)
Supplement: Supplementary file 1 — Supplementary material 1 (PDF 240 kb) [file 13238_2020_731_MOESM1_ESM.pdf]

Supplementary Information

Structure of African swine fever virus p15 reveals its dual role for membrane-association and DNA binding

Dan Fu*, Dongming Zhao*, Wei Zhang*, Guangshun Zhang, Mingyu Li, Zheng Zhang, Yuhui Wang, Dongdong Sun, Peng Jiao, Cheng Chen#, Yu Guo#, Zihe Rao

State Key Laboratory of Medicinal Chemical Biology and College of Pharmacy, Nankai University, Tianjin 300350, People's Republic of China

State Key Laboratory of Veterinary Biotechnology, Harbin Veterinary Research Institute, Chinese Academy of Agricultural Sciences, Harbin 150001, People's Republic of China.

School of Life Sciences, Tianjin University, Tianjin, 300071, People's Republic of China.

* These authors contributed equally to this paper;

# Correspondence and requests for materials should be addressed to Y. G and C.C (email: guoyu@nankai.edu.cn; chengchen@tju.edu.cn).

Keywords: ASFV, p15, crystal structure, membrane association, DNA binding

Running title: Crystal structure of ASFV p15

Contents

MATERIALS AND METHODS

Supplementary Table 1

MATERIALS AND METHODS

Expression and purification. The codon-optimized wild-type cDNA of ASFV p15 (GenBank ID: MK128995.1) is synthesized by GENEWIZ. The full-length p15 is cloned into a modified pMT plasmid with C terminal 6×His-tag. The cloning primers sequence are as follows: forward: 5'-CGCCATGGCCTTCAAACATGAAGCAATTCTGTAAGAT-3', reverse: 5'-GACTCGAGGTTACCGCCACCTTCCTTCTT- 3'. The resulting ASFV p15 expression plasmid along with a hygromycin resistance plasmid (pCoHygro) are used to obtain stably transfected S2 cells. Cells are grown to 4-10$\times 10^6$ cells ml^{-1} in ESF921 medium and expression was induced with 0.5 mM $CuSO_4$. After 4 days, the expression supernatant is collected through centrifugation. The recombinant ASFV p15 proteins are purified preliminarily on a Ni-NTA affinity column. ASFV p15 protein is further purified by Superdex 75 16/60 size-exclusion column in 20 mM HEPES, 150 mM NaCl, pH7.5. Fractions are pooled and concentrated to 10 mg/ml for crystallization. The ASFV p15 selenium derivative protein is also expressed in stably transfected S2 cells. Cells culture is in the△ESF921 methionine-free medium. 200 mg L-SeMet and 1 mM $CuSO_4$ are added to express the Se-ASFV p15 proteins. The purification of Se-ASFV p15 protein is the same as the native protein.

Crystallization. Initial crystallization conditions were screened by the sitting-drop vapour-diffusion method using commercial crystal screening kits (Hampton Research) at 16 ℃. The ASFV p15 protein and reservoir solutions were mixed in a ratio of 1:1, and all conditions were equilibrated against 100 μL of reservoir solution in a 48-well format plate. The small crystals appeared in the condition (2.0 M Ammonium sulfate, 0.08 M BIS-TRIS propane PH 7.0, 0.22 M Sodium malonate PH 7.0, 0.02 M HEPES PH 7.0, 0.1% v/v Jeffamine ED-2001 PH 7.0) after one week. Crystals for data collection are optimized by the hanging-drop vapour-diffusion method in the same

condition. Crystals of the Se-ASFV p15 appeared in the same condition after 8 days and grew to full size in 2 weeks at 16℃. Crystals were cryoprotected in crystallization solution with 40% PEG3350 and cooled in a dry nitrogen stream at 100 K for X-ray data collection.

X-ray data collection and structure determination. The data are collected at beamline BL17U1 and BL19U1 (SSRF, China) under cryogenic conditions at 100 K. The data sets were processed using the HKL3000. The SAD data phases are calculated and substantially improved by solvent flattening using the PHENIX program (Adams et al., 2002). A model is manually built into the modified experimental electron density using COOT (Emsley and Cowtan, 2004) and further refined in PHENIX. The model geometry is verified using the program MolProbity (Chen et al., 2009). Structural figures are drawn using the program PyMOL (http://www.pymol.org).

Analytical ultracentrifugation (AUC) assay. AUC experiments are performed at 4℃ in a Beckman Optima XL-I, operating under velocity sedimentation (SV) mode using an AN-50 Ti rotor with two-channel charcoal-filled centrepieces. Sedimentation velocity experiments were performed at 40,000 rpm loading 1 mg/ml protein. Data were collected at 280 nm in a continuous mode and fitted with SEDFIT.

Mutant protein production. The key residues K126, E87, T105, N108, C8, C29, C29+C8 and Y62 are mutated to alanine to explore the role on the formation of dimer and hexamer. All the residues in the positively charged patches of the p15 protein hexamer side A (K5+K49+K52+K104+K126) and side B (K9+K38+R131+R132) are all mutated to alanines. The site-directed mutations of p15 were generated by using the Fast Mutagenesis System (TransGen Biotech, China). The sequence of all the mutation constructions was verified by DNA sequencing (GENGWIZ, China). All the mutants protein are expressed and purified following the procedure of the wild-type

protein. The AUC elution profiles of the side A/B mutant are as similar as the wild-type protein, indicating that these mutations did not interfere with the overall structures.

Electrophoretic Mobility Shift Assays (EMSA). The DNA segments (the first 25 bps in the ASFV genome, 5- ATATACCATATTATTGCTATTGCCA-3') are annealed to form a DNA duplex. The binding reaction contained dsDNA (5 μg), 20 mM HEPES, 150 mM NaCl, PH 7.5 and gradient purified proteins while the control group had no protein. After incubation at 16°C for 4 hours, 1.5 μl of 10× colourless DNA loading buffer was added. The reaction mixture was then analyzed by 1% agarose gel in 1×TAE buffer (40 mM Tris, 2 mM EDTA), and DNA was visualized by superRed (biosharp) staining.

Liposome floatation experiments. Pre-formed liposomes of different PC (1-palmitoyl-2-oleyl-sn-glycero-3-phosphocholine), PS (1, 2-dioleyl-sn-glycero-3-phospho-L-serine) composition and cholesterol are dissolved in chloroform and dried under a nitrogen stream and Vacuum concentrator. Then, the resuspended pre-formed liposomes in a HEPES buffer are diluted to a final lipid concentration of 10mM. Large unilamellar vesicles (LUVs) were made by 10 freeze-thaw cycles in liquid nitrogen. LUVs were then passed through a polycarbonate membrane with 100 nm pore size (Avanti Polar Lipids). The type of different liposomes used in the investigation are 100 nm PS: PC: cholesterol (10:40:50 mol %) liposomes, 100 nm PS: PC: cholesterol (25:25:50 mol %) liposomes and 100 nm PS: PC: cholesterol (25:25:50 mol %) liposomes.

Liposome association is assayed using a discontinuous Accudenz gradient. Liposomes (1 mM lipid concentration) are separately incubated with p15 wild-type protein or mutants proteins at a 100 lipid: protein molar ratio in the HEPES buffer overnight at 4℃. After incubation, 225 μl of the protein/liposome mixture is added to 225 μl 80% (w/v) Nycodenz solution, bringing it to a 40% (w/v) Nycodenz solution. The mixture

is placed into a 5x41 mm Ultra-Clear tube (Beckman) overlaid with 375 μl 30% (w/v)

**Parameter**	**Se-p15**	**Native-p15**

Nycodenz solution in the HEPES buffer and then covered with 75 μl HEPES buffer without glycerol. The gradient is centrifuged in a Sw-55 Ti rotor for 5 hours at 48,000 rpm at 4℃. A total of nine fractions are collected and samples are run on an SDS-PAGE and Coomassie-stained.

Data accessibility. Atomic coordinates and structure factors for the protein of ASFV p15 has been deposited in the Protein Data Bank under accession number 7BQ9.

Reference

Adams, P.D., Grosse-Kunstleve, R.W., Hung, L.-W., Ioerger, T.R., McCoy, A.J., Moriarty, N.W., Read, R.J., Sacchettini, J.C., Sauter, N.K., and Terwilliger, T.C. (2002). PHENIX: building new software for automated crystallographic structure determination. Acta Crystallographica Section D Biological Crystallography 58, 1948-1954.

Chen, V.B., Arendall, W.B., Headd, J.J., Keedy, D.A., Immormino, R.M., Kapral, G.J., Murray, L.W., Richardson, J.S., and Richardson, D.C. (2009). MolProbity: all-atom structure validation for macromolecular crystallography. Acta Crystallographica Section D Biological Crystallography 66, 12-21.

Emsley, P., and Cowtan, K. (2004). Coot: model-building tools for molecular graphics. Acta Crystallographica Section D Biological Crystallography 60, 2126-2132.

PDB accession no.		7BQ9
X-ray source	SSRF BL19U1	SSRF BL17U1
Wavelength (Å)	0.9792	1.0715
Space group	$P2_13$	$P2_13$
Unit cell parameters (Å; °)	a=b=c=116.737	a=b=c=116.737
	α=β=γ= 90	α=β=γ= 90
Resolution range (Å)	30.00-3.50(3.56-3.50)	50.00-2.60 (2.76-2.60)
No. of unique reflections	7247 (334)	16329 (1580)
Completeness (%)	100.0 (100.0)	99.63 (96.60)
Redundancy	39.1 (38.0)	40.2 (29.6)
I/σ(I)	33.25 (3.0)	30.7 (1.0)
Rmerge (%)	15.4 (219.9)	10.1 (290.9)
CC1/2	1.00 (0.73)	1.00 (0.51)
**Refinement statistics**		
Resolution range (Å)		47.66 - 2.61 (2.71 - 2.61)
No. of reflections used in refinement		16313 (1564)
No. of reflections used for Rfree		1629 (158)
Rwork (%)		18.12 (34.35)
Rfree (%)		21.81 (36.10)
No. of nonhydrogen atoms		2408
Protein		2408
Solvent		0
Avg B-factors		94.11
Protein		94.11
Solvent		0
RMSD		
Bond length (Å)		0.014
Bond angle (°)		1.12
MolProbity clash score		7.81
Ramachandran (%)		
Favoured		96.03
Allowed		3.97
Outliers		0.00

Supplementary Table1 | Data collection and refinement statistics*

*Numbers in the brackets are for the highest resolution shell. $R_{merge} = \Sigma_h\Sigma_l \mid I_{ih} - \langle I_h \rangle \mid / \Sigma_h\Sigma_I \langle I_h \rangle$, where $\langle I_h \rangle$ is the mean of the observations I_{ih} of reflection h. $R_{work} = \Sigma(\|F_p(obs)| - |F_p(calc)\|) / \Sigma|F_p(obs)|$; R_{free} is an R factor for a pre-selected subset (5%) of reflections that was not included in refinement.
